# Fatal Accelerated Cirrhosis after Imported HEV Genotype 4 Infection

**DOI:** 10.3201/eid2109.150300

**Published:** 2015-09

**Authors:** Ryan B. Perumpail, Aijaz Ahmed, John P. Higgins, Samuel K. So, J. Lynn Cochran, Jan Drobeniuc, Tonya R. Mixson-Hayden, Chong-Gee Teo

**Affiliations:** Stanford University School of Medicine, Palo Alto, California, USA (R.B. Perumpail, A. Ahmed, J.P. Higgins, S.K. So);; Birmingham Gastroenterology Associates, Birmingham, Alabama, USA (J.L. Cochran);; Trinity Medical Center, Birmingham (J.L. Cochran);; Centers for Disease Control and Prevention, Atlanta, Georgia, USA (J. Drobeniuc, T.R. Mixson-Hayden, C.-G. Teo)

**Keywords:** hepatitis E, HEV, genotype 4, chronic liver disease, acute liver disease, cirrhosis, Hong Kong, liver transplantation, viruses

**To the Editor:** Hepatitis E is a viral hepatitide that is endemic in many developing countries. In its classic form, it results from ingesting fecally contaminated water that carries hepatitis E virus (HEV), and it frequently resolves without treatment. When hepatitis E is imported to the United States, it originates mainly from persons who have acquired HEV genotype 1 infection from South Asia (*1*). We report imported HEV genotype 4 infection (Technical Appendix Figure, panel A) in a patient during which cirrhosis and fatal hepatic decompensation ensued.

The patient was a 68-year-old man of Chinese ethnicity who had been a California resident since 1985. He sought treatment for mild jaundice in April 2013 in Hong Kong, where he had been staying for 7 weeks. Sixteen years before, he had undergone orthotopic liver transplantation at Stanford University Medical Center (Palo Alto, California, USA) for hepatitis B cirrhosis. Since then, he had received entecavir and tacrolimus for maintenance and had been vaccinated against hepatitis A virus. Until his current illness, routine liver function tests had not indicated hepatic dysfunction (values in November 2012: alanine aminotransferase 2 IU/L, aspartate aminotransferase 24 IU/L, alkaline phosphatase 67 IU/L, total bilirubin 0.5 mg/dL).

When the patient returned to the United States, 3 weeks after onset of jaundice, the initial work-up showed the following values: alanine aminotransferase 149 IU/L, aspartate aminotransferase 59 IU/L, alkaline phosphatase 193 IU/L, total bilirubin 2.8 mg/dL (Technical Appendix Figure, panel B). Hepatitis B virus DNA and antinuclear antibodies were not detected, and the tacrolimus level was stable. Ultrasound revealed a normal transplanted liver. A liver biopsy specimen showed mild portal, biliary, and lobular inflammation and early biliary injury (Figure, panels A, B). The prednisone dosage was escalated, and mycophenolate mofetil was added. Liver enzyme activity showed some improvement, but the bilirubin level continued to rise (Technical Appendix Figure, panel B).

**Figure:**
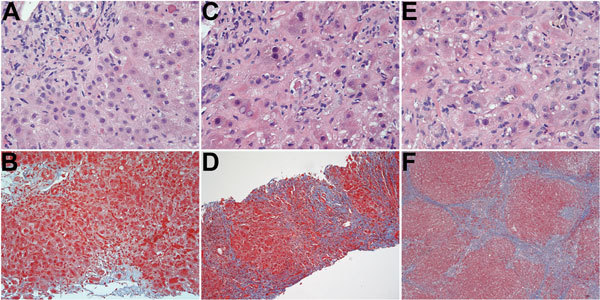
Serial histologic changes in liver of the patient who received a diagnosis of hepatitis E after a visit to Hong Kong in 2013 (A and B: at first biopsy; C and D: second biopsy; E and F: third biopsy. A) Mild mixed portal infiltration; minimal lobular inflammation; acidophil body present at upper right; and bile duct showing injury with lymphocytic infiltration (original magnification ×400). B) Mild portal inflammation; some interface activity; and portal tracts not showing increased fibrosity (original magnification ×200). C) Mononuclear infiltration of portal tract at upper right with bile duct/ductular infiltration and injury; lobular changes more severe, showing more inflammation, acidophil bodies and reactive nuclear change in hepatocytes with ballooning of some hepatocytes (original magnification ×400). D) Portal and lobular inflammation; and marked increase in fibrosis with bridging and regenerative nodule formation (original magnification ×100). E) Extensive lobular inflammation and reactive hepatocytic changes with nuclear enlargement, prominent nucleoli, and ballooning (original magnification ×400). F) Well-developed cirrhosis (original magnification ×40). Hematoxylin and eosin staining (A, C, E); Masson trichrome staining. (B, D, F).

A biopsy specimen taken 3 months later showed grade 3 hepatitis with bile ductular reaction, bridging hepatocytic necrosis and fibrosis, and regenerative nodule formation (Figure, panels C, D). A blood sample taken about this time tested positive for HEV RNA. The patient was then given ribavirin (1,000 mg/d). Before hepatitis E was diagnosed, tacrolimus was given (1 mg 2×/d); when the diagnosis was confirmed, the tacrolimus dose was reduced to 0.5 mg every other day. Four months after the patient sought treatment, ascites was noted. Ribavirin was stopped because of pancytopenia. Blood samples subsequently tested negative for HEV RNA, but HEV IgM and IgG were found. Hepatic function did not improve.

Eight months after onset of the patient’s condition, marked hepatic decompensation occurred (Technical Appendix Figure), culminating in esophageal variceal hemorrhage. The patient was placed on a waiting list and then underwent liver transplantation, but he died during the operation from complications of hemorrhage. Biopsy of the liver explant revealed intense lobular inflammation with the hepatocellular reactive changes persisting and stage IV fibrosis (Figure, panels E, F).

The patient had lived and worked in Hong Kong before he became a resident of the United States. He had not visited Hong Kong in the 3 years preceding his most recent trip, nor had he traveled to Europe. Review of his medical records revealed no evidence of hepatic dysfunction after his previous travels. Considering that his most recent visit to Hong Kong coincided with the incubation period of hepatitis E (*2*), he most likely acquired HEV genotype 4 infection during that visit. 

In China over the past decade, national notifications of HEV infection have risen, with 28,232 cases reported in 2013 (*3*). In Hong Kong, where a rising trend in hepatitis E notifications also has been observed (150 cases reported in 2012 [*4*]), HEV infections are almost all associated with HEV genotype 4 (*5*). 

This patient’s HEV subgenomic sequence was closely related to human and porcine HEV genotype 4 sequences reported from mainland China and Hong Kong (Technical Appendix Figure, panel A). Porcine liver has been implicated as a possible HEV transmission vehicle in that region (*6*); although we do not know whether the patient ate food that carried HEV, the possibility underscores the importance of avoiding eating inadequately cooked animal-derived food products during international travel (*2*).

Chronic hepatitis with accelerated cirrhosis has been reported in solid-organ transplant recipients infected with HEV genotype 3, but not with genotype 4 (*7*). Serial liver biopsy specimens from the patient showed persistent and worsening hepatitis and rapid onset of fibrosis that intensified (Technical Appendix Figure, panel B). 

Testing for HEV infection is recommended during initial assessments of posttransplant hepatic dysfunction because histologic appearances in liver biopsy specimens may not clearly distinguish between graft rejection and acute viral hepatitis (Figure, panels A, B). Early diagnosis of hepatitis E should lead to prompt administration of antiviral therapy and appropriate adjustments to the immunosuppressant drug regimen, particularly because some drugs can exert opposing effects on HEV replication (*8*).

**Technical Appendix.** A) Phylogenetic tree comparing a 258-nt sequence within hepatitis E virus (HEV) open reading frame 1 (*1*) of the patient who visited Hong Kong in 2013 with corresponding, representative GenBank sequences. Included is a corresponding sequence from patient A, a 63-year-old Caucasian man, a resident of Alabama, in whom acute, self-resolving hepatitis developed 5 weeks after he returned from a 2-week visit to Shandong, China. Numerals beside each sequence denote year of sequence reporting; alphanumerics denote GenBank accession numbers. GT, genotype; CH, China; HK, Hong Kong; MX, Mexico; US, United States. B) Chronology of changes in liver function and hepatitis E markers in the patient.
