# Supplementary material for: Fatal Accelerated Cirrhosis after Imported HEV Genotype 4 Infection
Source: Emerg Infect Dis. 2015 Sep;21(9):1679–81. doi: 10.3201/eid2109.150300 (PMC4550159; doi:10.3201/eid2109.150300)
Supplement: Supplementary file 1 — Technical Appendix. A) Phylogenetic tree comparing a 258-nt sequence within hepatitis E virus (HEV) open reading frame 1 (1) of the patient who visited Hong Kong in 2013 with corresponding, representative GenBank sequences. Included is a corresponding sequence from patient A, a 63-year-old Caucasian man, a resident of Alabama, in whom acute, self-resolving hepatitis developed 5 weeks after he returned from a 2-week visit to Shandong, China. Numerals beside each sequence denote year of sequence reporting; alphanumerics denote GenBank accession numbers. GT, genotype; CH, China; HK, Hong Kong; MX, Mexico; US, United States. B) Chronology of changes in liver function and hepatitis E markers in the patient. [file 15-0300-Techapp-s1.pdf]

Article DOI: http://dx.doi.org/10.3201/eid2109.150300

Fatal Accelerated Cirrhosis after Imported HEV Genotype 4 Infection

Technical Appendix

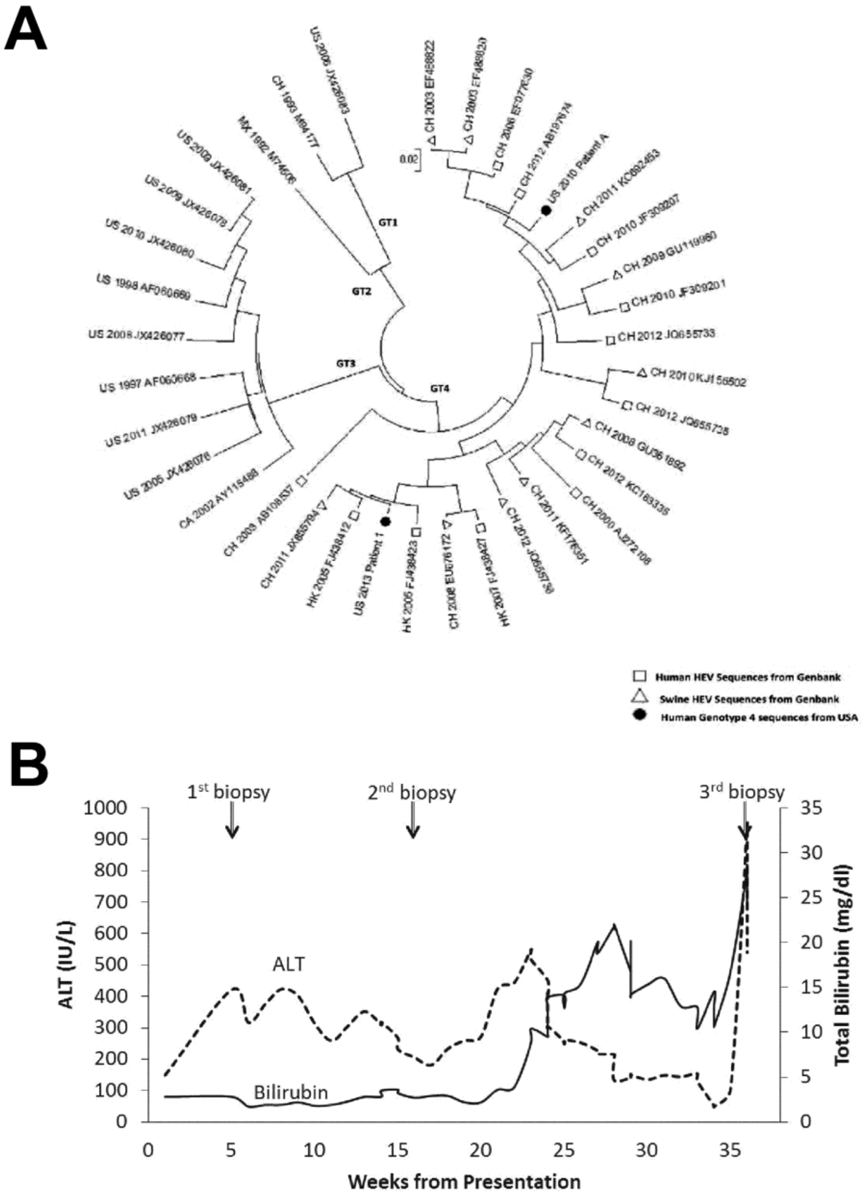

Technical Appendix Figure. A) Phylogenetic tree comparing a 258-nt sequence within hepatitis E virus (HEV) open reading frame 1 of the patient who visited Hong Kong in 2013 with corresponding, representative GenBank sequences. Included is a corresponding sequence from patient A, a 63-year-old Caucasian man, a resident of

Alabama, in whom acute, self-resolving hepatitis developed 5 weeks after he returned from a 2-week visit to Shandong, China. Numerals beside each sequence denote year of sequence reporting; alphanumerics denote GenBank accession numbers. GT, genotype; CH, China; HK, Hong Kong; MX, Mexico; US, United States. B) Chronology of changes in liver function and hepatitis E markers in the patient.
